# Risk prediction of dysthyroid optic neuropathy based on CT imaging features combined the bony orbit with the soft tissue structures

**DOI:** 10.3389/fmed.2022.936819

**Published:** 2022-08-24

**Authors:** Shengnan Cheng, Yangcan Ming, Mang Hu, Yan Zhang, Fagang Jiang, Xinghua Wang, Zefeng Xiao

**Affiliations:** ^1^Department of Ophthalmology, Wuhan Hospital of Traditional Chinese and Western Medicine, Wuhan, China; ^2^Department of Pediatrics, Wuhan Hospital of Traditional Chinese and Western Medicine, Wuhan, China; ^3^Department of Ophthalmology, Union Hospital, Tongji Medical College, Huazhong University of Science and Technology, Wuhan, China

**Keywords:** dysthyroid optic neuropathy, risk prediction, CT imaging, bony orbit, soft tissue structures

## Abstract

**Purpose:**

To analyze computed tomographic (CT) imaging features of patients with dysthyroid optic neuropathy (DON) retrospectively and deduce a more appropriate predictive model.

**Methods:**

The CT scans and medical records of 60 patients with clinically proven Graves' ophthalmopathy (GO) with (26 women and 10 men) and without DON (16 women and 8 men) were retrospectively reviewed, and 20 age- and sex-matched control participants (12 women and 8 men) were enrolled consecutively. The bony orbit [orbital rim angle (ORA), medial and lateral orbital wall angles (MWA and LWA), orbital apex angle (OAA), and length of the lateral orbital wall (LWL)], and the soft tissue structures [maximum extraocular muscle diameters (Max EOMD), muscle diameter index (MDI), medial and lateral rectus bulk from inter-zygomatic line (MRIZL and LRIZL), proptosis, intraorbital optic nerve stretching length (IONSL), superior ophthalmic vein diameter (SOVD), apical crowding, and presence of intracranial fat prolapse] were assessed on a clinical workstation. The CT features among groups were compared, and a multivariate logistic regression analysis was performed to evaluate the predictive features of DON.

**Results:**

All bony orbital angle indicators, except ORA (*p* = 0.461), were statistically different among the three groups (all *p* < 0.05). The values of MWA, LWA, OAA, and LWL were larger in the orbits with the DON group than in the orbits without the DON group (all *p* < 0.05). The MDI, MRIZL, proptosis, IONSL, and SOVD were statistically significantly different among the three groups (all *p* < 0.05), in which the orbits with the DON group were significantly higher than the orbits without the DON group and control group. The apical crowding was more severe in the orbits with the DON group than in the orbits without the DON group (*p* = 0.000). There were no significant differences in the LRIZL and the presence of intracranial fat prolapse (all *p* > 0.05). The multivariate regression analysis showed that the MWA, MDI, and SOVD were the independent factors predictive of DON. The sensitivity and specificity for the presence of DON by combining these three indicators were 89 and 83%, respectively.

**Conclusion:**

Bone and soft tissue CT features are useful in the risk prediction of DON, especially the MWA, MDI, and SOVD were the independent factors predictive of DON.

## Introduction

Graves' ophthalmopathy (GO) is the most common extra-thyroidal manifestation of Graves' disease (GD), with approximately 40% of patients with GD presenting with GO. Graves' ophthalmopathy may occur in patients with hyperthyroidism, euthyroidism, or hypothyroidism (1–3). The disease usually begins to present itself as a self-limiting active phase, lasting from 18 to 24 months, followed by an inactive phase (4). The tissue within the relatively fixed volume of the bony orbit was expanded by the inflammation, the accumulation of mucopolysaccharides, and the increased fat content. The diagnosis of GO is usually made clinically. Graves' ophthalmopathy has a wide range of sequelae, including early appearance changes and subsequent intractable diplopia, as well as late visual impairment and even corneal perforation due to deteriorating exposed ulcers (2). Ocular signs and symptoms during the GO activity phase may manifest as eyelid retraction, conjunctival congestion, chemosis, proptosis, diplopia, corneal ulcers, and the relatively rare dysthyroid optic neuropathy (DON).

Dysthyroid optic neuropathy has long been considered the most horrible ocular complication of GO, affecting 4–8% of the patients, which could cause severe visual impairment and even blindness (5). The mechanism of DON is currently unclear, and the mechanical compression was the most widely accepted account, that is, the optic nerve at the orbital apex is compressed by the enlarged extraocular muscle (EOM) (6). Dysthyroid optic neuropathy may present multifarious symptoms and signs (7), so its diagnosis relies on several clinical features, such as decreased best corrected visual acuity, defect of visual fields, defective color vision, impaired brightness perception, abnormal visually evoked potential, relatively afferent pupillary defects, and edema or atrophy of the optic nerve head (5). We often assume DON when one of these features is present and no other cause accounts for the defect in patients with GO, but visual impairment in patients with GO is commonly related to other factors (8). Accordingly, misleading results may occur following direct optic nerve function testing in patients with GO, which occasionally makes it difficult to distinguish probable DON from definitive DON. Furthermore, DON can progress rapidly and perform obviously, or make slow progress and perform insidiously, so it is easy to be misdiagnosed and missed diagnosed. It can cause blindness when it progresses to an advanced stage, seriously affecting the quality of life. Therefore, an accurate and timely diagnosis is essential to reduce the incidence of DON and improve the prognosis.

The diagnosis of DON is mainly based on clinical findings and imaging findings, such as computed tomography (CT), magnetic resonance imaging (MRI), ultrasonography, and color Doppler imaging. A previous study set CT scan to be the preferred imaging technique for detecting DON (9–14). Imaging can confirm possible EOM involvement and may help determine whether the patients with GO are in the early acute inflammatory stage or the inactive fibrotic stage (15). Imaging is of great help in identifying patients who are prone to develop DON. It enables timely diagnosis of DON and better monitoring of the treatment effects, thus avoiding permanent visual impairment or even blindness (16). The diagnosis of DON is associated with various imaging features, including the extent of enlargement of EOM, the increased volume of orbital fat, the extent of orbital apex crowding, and the presence of intracranial fat prolapse (16–20). Studies have shown that the role of the bony orbit in the onset of DON cannot be ignored (21). Although previous studies have evaluated the role of EOM thickening and increased orbital soft tissue volume in the DON pathophysiology, to the best of our knowledge, there have been few studies on the role of the bony orbit and its combined orbital soft tissue structures in DON. This study mainly concerns analyzing the bony orbital structure and soft tissue CT features relating to DON in GO and their role in the risk prediction of DON occurrence.

## Materials and methods

### Ethical approval

This case series study is retrospective. We collected and reviewed the medical records of the patients affected by GO with and without DON between 2013 and 2019 and visited the Department of Ophthalmology, Union Hospital, Tongji Medical College, and Huazhong University of Science and Technology. This study was approved by the Ethics Committee of Union Hospital affiliated with Tongji Medical College, Huazhong University of Science and Technology, Wuhan, China. It was conducted in strict accordance with the Helsinki Declaration. Written informed consent was provided by all participants. We conducted the sample size calculation by using the PASS software version 15 (AQ5, NCSS, LLC, Kaysville, UT, USA), with 90% power, 0.05 alpha, and 0.50 effect size. It was carried out prior to the study, and the minimum sample size was 63 after calculation.

### Methods

The diagnosis of GO was based on the Bartley criteria (22). The diagnosis of DON relies on any combination of visual deficits, including visual acuity, visual field, and color vision. At least one of the following conditions was supported: apical crowding, optic disc edema, and relative afferent pupil defect. Optic nerve dysfunction alone was responsible for all deficits (5, 6). Patients with prior history of decompression surgery, medical treatment, or other ophthalmic conditions that could alter the orbital anatomy were excluded. Exclusion criteria also included any ocular disease affecting vision function, such as highly myopia or hyperopia, amblyopia, corneal ulcer, obvious refractive interstitial opacity, various types of glaucoma, retinal disease, and optic neuropathy. The diagnosis of glaucoma was based on a definite medical history and reliable clinical examination, which occurred before the definite diagnosis of GO and were not related to GO. As for the optic neuropathy referred to above, we excluded several patients with optic neuritis, ischemic optic neuropathy, and traumatic optic neuropathy. For comparison, we included 20 age- and gender-matched patients without GO who underwent CT in our hospital for one eye trauma, orbital fracture, or orbital tumor, and we included their healthy eyes as the control group.

The orbital CT scans were part of a routine clinical examination for patients with GO on helical CT scanners in our institution, with acquisition parameters of 120 kilovolt (peak) (kVp), 200 mA, 160 mm display field of view (FOV), and pitch of 0.8–1.0. The axial scans were contiguous 3 mm sections parallel to the infra-orbitomeatal line, and coronal scans were reconstructed orthogonal to this plane. Two radiologists, who had 5–10 years of experience in orbital imaging, analyzed each orbital CT image. Aware of the diagnosis of GO and DON but blinded to the medical history details, the two researchers construed the image features by consensus. The two researchers would jointly review the images to reach a consensus for further analysis if there was a disagreement. Finally, inter-observer and intra-observer concordance was confirmed to be above 90%. The electronic calipers were available on routine clinical reporting workstations to carry out angular and linear measurements and to quantitatively assess the CT features of bony orbital confines and soft tissue structures.

Measuring standardized orbital angles on axial scans were used to quantitatively evaluate the capacity of the bony orbit. The indicators of the bony orbit were measured as follows: (1) orbital rim angle (ORA) (measured on the section containing the medial canthus ligament at the axial plane), (2) medial and lateral orbital wall angles (MWA and LWA) [measured on the section containing the widest diameter of the medial and lateral rectus muscles at the axial plane, and the angular change was recorded referring to the ORA, which has a positive value (wider than the ORA) and a negative value (narrower than the ORA)], (3) orbital apex angle (OAA) (subtended by the medial and lateral orbital walls at the axial plane, which was defined as the anterolateral border of the groove in the sphenoid body formed by the terminal intra-cavernous portion of the internal carotid artery), and (4) length of the lateral orbital wall (LWL) (measured as the straight-line distance along the lateral wall from the lateral border of the superior ophthalmic fissure to the orbital rim at axial plane).

The indicators of the soft tissue structures were measured as follows: (1) maximum extraocular muscle diameter (max EOMD) [measured as the maximum diameters of the medial and lateral recti, the inferior rectus and superior muscle group, and the superior oblique muscle on axial and coronal images (20)], (2) muscle diameter index (MDI) (computed by the sum of the above EOM diameters), (3) medial and lateral rectus bulk from inter-zygomatic (IZ) line (MRIZL and LRIZL) (measured as the perpendicular distance from the midpoint of the maximum medial and lateral rectus muscles diameter to the IZ line at axial plane), (4) proptosis (measured as the vertical distance from the corneal vertex to the IZ line), (5) intraorbital optic nerve stretching length (IONSL) (measured as the straight-line distance from the orbital apex point to the retrobulbar optic nerve at axial plane), (6) superior ophthalmic vein (SOV) diameter (SOVD) [measured on axial images according to the method described by Nugent et al. (20)], (7) apical crowding [graded on a four-grade scale at coronal images (20), specifically, no effacement of perineural fat as Grade 0, mild (1–25%) effacement of perineural fat as Grade 1, moderate (25–50%) effacement of perineural fat as Grade 2, and severe (50%) effacement of perineural fat as Grade 3], and (8) presence of intracranial fat prolapse [evaluated as present or absent at the axial plane by using the method described by Birchall et al. (19)].

### Statistical analysis

Data analysis was performed by using SPSS for Windows, version 20 (SPSS Inc., Chicago, IL, USA). The normality of the distribution was tested with Shapiro-Wilk statistics. The Mann–Whitney *U*-test, independent *t*-test, and chi-square test were used when necessary. A Bonferroni correction was applied for multiple comparisons. All data were presented as the mean ± SD. Spearman's correlation coefficients were conducted to correlate the bony orbital parameters to the corresponding soft tissue structural parameters. A multivariate logistic regression analysis was conducted to identify the predictive factors for the occurrence of DON. A receiver operating characteristic curve (ROC) analysis was conducted to calculate the sensitivity and specificity of the predictive factors. A *p*-value of <0.05 was considered statistically significant.

## Results

We consecutively enrolled CT scans and medical records of 60 patients with clinically proven GO with (36 eyes; 26 women and 10 men; mean age, 50.33 ± 9.40 years; range, 28–71 years) and without DON (24 eyes; 16 women and 8 men; mean age, 49.33 ± 10.66 years; range, 28–71 years). We also enrolled 20 control participants (20 eyes; 12 women and 8 men; mean age, 49.05 ± 11.18 years; range, 29–68 years). Age and gender did not significantly differ among the groups (both *p* > 0.05).

**Figure 1:**
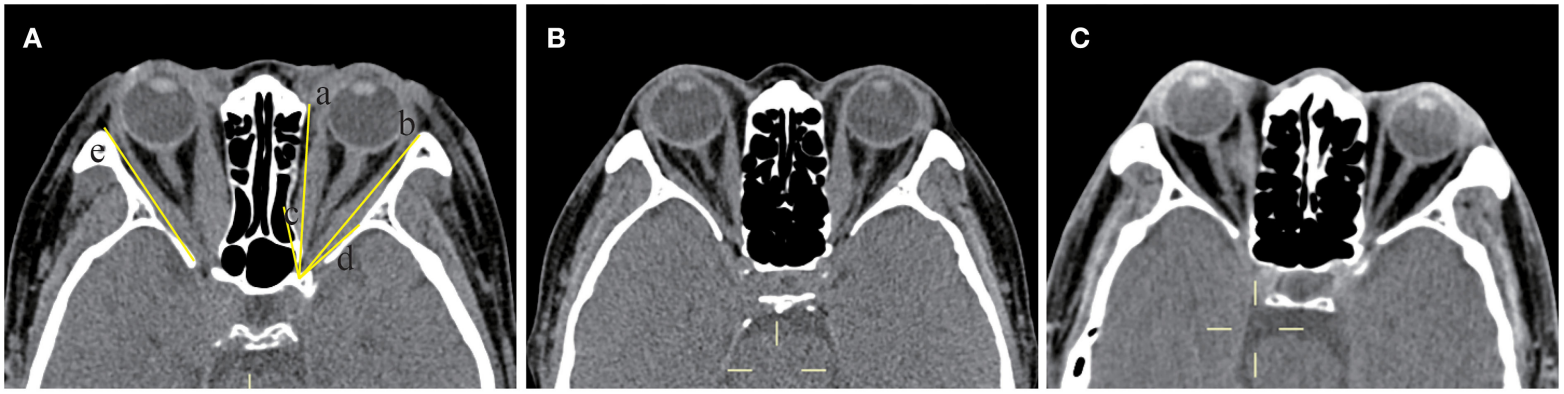
Computed tomography (CT) images at the axial section of bony orbital angles measurement. **(A)** The orbit with DON, which labels the measurement of the bony orbital angles. The orbital rim angle (ORA) was measured at the level of the medial canthus ligament, measuring the angle (degrees) between two lines, with line a drawn along the margin of the medial orbital wall and line b connecting the frontozygomatic process and the most medial portion of the inner border of the sphenoid wing with extension intracranially until it intercepts with line a. The same axial section containing the bulk of the medial and lateral rectus muscles showed the medial wall angle (MWA) between lines a and c, the lateral wall angle (LWA) between lines b and d, and the orbital apex angle (OAA) between lines c and d. The length of the lateral orbital wall (LWL) was measured on the section just inferior to the anterior clinoid (labeled line e). **(B)** The orbit without DON. **(C)** The orbit of the normal control (right eye).

In the comparison of bony orbital angles (Figure 1; Table 1), all indicators, except ORA (*p* = 0.461), were statistically different among the three groups (all *p* < 0.05). The MWA (3.98 ± 7.17 degrees vs. −0.25 ± 7.18 degrees), the LWA (6.71 ± 3.15 degrees vs. 4.53 ± 2.24 degrees), the OAA (51.26 ± 8.60 degrees vs. 44.05 ± 8.49 degrees), and the LWL (44.31 ± 2.71 vs. 42.89 ± 2.45 mm) were larger in the orbits with the DON group than in the orbits without the DON group (*p* = 0.024, 0.015, 0.001, 0.001, and 0.048, respectively), while there were no significant differences in the ORA (40.57 ± 2.97 vs. 39.77 ± 3.10 degrees) between the two groups (*p* = 0.097).

**Table 1:** Comparison of demographics, bony orbit, and soft tissue structures in three groups.

	**Orbits with DON (*n* = 36)**	**Orbits without DON (*n* = 24)**	**Controls (*n* = 20)**	*P* ^*^	P^**^(Orbits with DON vs. without DON)
**Demographics**					
Age	50.33 ± 9.40	49.33 ± 10.66	49.05 ± 11.18	0.888	0.786
Gender (female %)	72	67	60		0.645^‡^
**Bony orbital angle (degrees)**
ORA	40.57 ± 2.97	39.77 ± 3.10	39.78 ± 1.79	0.461	0.097
MWA	3.98 ± 7.17	−0.25 ± 7.18	−2.06 ± 1.90	0.002	0.024
LWA	6.71 ± 3.15	4.53 ± 2.24	4.74 ± 2.47	0.006	0.015
OAA	51.26 ± 8.60	44.05 ± 8.49	42.46 ± 2.99	0.000	0.001^†^
LWL (mm)	44.31 ± 2.71	42.89 ± 2.45	42.40 ± 1.81	0.013	0.048
**Max EOMD (mm)**
Medial rectus	7.97 ± 2.43	4.95 ± 2.25	3.62 ± 0.75	0.000	0.000
Lateral rectus	4.51 ± 1.83	3.15 ± 1.11	2.47 ± 0.69	0.000	0.003
Superior muscle group	6.16 ± 2.65	4.70 ± 2.16	2.65 ± 0.72	0.000	0.044
Inferior rectus	6.70 ± 1.76	4.00 ± 2.19	2.70 ± 0.74	0.000	0.000
Superior oblique	2.54 ± 1.08	2.07 ± 0.96	1.35 ± 0.75	0.000	0.090
MDI	27.87 ± 5.65	18.87 ± 6.53	12.78 ± 2.25	0.000	0.000
MRIZL	16.92 ± 4.18	14.38 ± 3.50	15.13 ± 1.86	0.023	0.019^†^
LRIZL	18.22 ± 3.44	17.41 ± 1.98	18.24 ± 1.38	0.466	0.536
**Other indicators**					
Proptosis (mm)	21.12 ± 2.76	17.94 ± 3.39	14.88 ± 1.38	0.000	0.000^†^
IONSL (mm)	37.74 ± 2.77	35.59 ± 3.86	29.93 ± 2.76	0.000	0.006
SOVD (mm)	2.34 ± 0.70	1.71 ± 0.49	1.35 ± 0.50	0.000	0.000^†^
Apical crowding	2.19 ± 0.78	1.00 ± 0.91	0.00 ± 0.00	0.000	0.000
Presence of intracranial fat prolapse	18(50.0%)	10(41.7%)	5(40.0%)	0.196	0.526^‡^

*DON, dysthyroid optic neuropathy; ORA, orbital rim angle; MWA, medial wall angle; LWA, lateral wall angle; OAA, orbital apex angle; Max EOMD, maximum extraocular muscle diameter; MDI, muscle diameter index; MRIZL, medial rectus bulk from interzygomatic line; LRIZL, lateral rectus bulk from interzygomatic line; IONSL, intraorbital optic nerve stretching length; SOVD, superior ophthalmic vein diameter. Data are presented as mean ± standard deviation (SD). Statistical significance is defined at p <0.05*.

* *Obtained by the Kruskal-Wallis test*.

** *Mann–Whitney U-test or otherwise, as indicated*.

† *Independent t-test*.

‡ *Chi-square test*.

**Figure 2:**
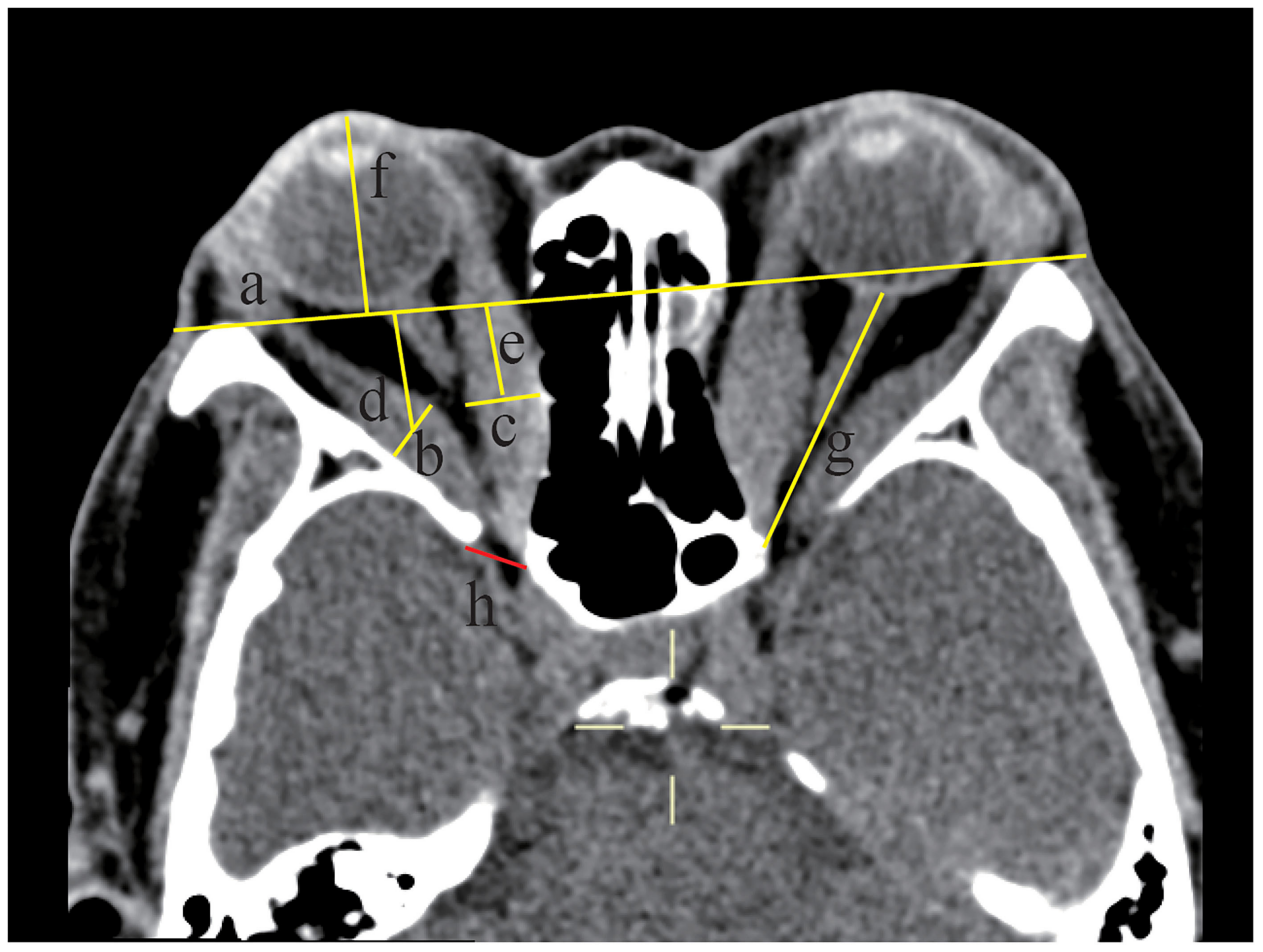
Soft tissue structures were measured by computed tomography (CT) at the axial section of a 58-year-old female dysthyroid optic neuropathy (DON) patient. The axial section at the midglobe level showed the interzygomatic line (IZ) (labeled line a) and maximum horizontal diameters of the right lateral rectus muscle (labeled line b and the length was 9.11 mm) and the medial rectus muscle (labeled line c and the length was 10.48 mm). The distance from the midpoint of the maximum muscular diameter of the lateral rectus muscle (labeled line d and the length was 17.47 mm) and medial rectus muscle (labeled line e and the length was 13.88 mm) to the IZ was recorded. Proptosis of the right eyeball was measured from the center of the anterior cornea to the IZ (labeled line f and the length was 24.66 mm). The optic nerve stretch of the left eyeball was measured from the retrobulbar optic nerve to the orbital apex point (labeled line g and the length was 36.21 mm). Intracranial fat prolapse was present in the right eye (labeled h), and the red line connected the most inner border of the sphenoid wing and the most anterior border of the sphenoid body groove.

In the comparison of soft tissue structures (Figure 2; Table 1), the MDI (27.87 ± 5.65 vs. 18.87 ± 6.53 vs. 12.78 ± 2.25 mm), the MRIZL (16.92 ± 4.18 vs. 14.38 ± 3.50 vs. 15.13 ± 1.86 mm), the proptosis (21.12 ± 2.76 vs. 17.94 ± 3.39 vs. 14.88 ± 1.38 mm), the IONSL (37.74 ± 2.77 vs. 35.59 ± 3.86 vs. 29.93 ± 2.76 mm), and the SOVD (2.34 ± 0.70 vs. 1.71 ± 0.49 vs. 1.35 ± 0.50 mm) were statistically significant among the three groups (*p* = 0.000, 0.023, 0.000, 0.000, and 0.000, respectively), which were the largest in the orbits with the DON group. The apical crowding was more severe in the orbits with the DON group than in the orbits without the DON group (2.19 ± 0.78 vs. 1.00 ± 0.91, *p* = 0.000). There were no significant differences in the LRIZL and the presence of intracranial fat prolapse (all *p* > 0.05).

**Figure 3:**
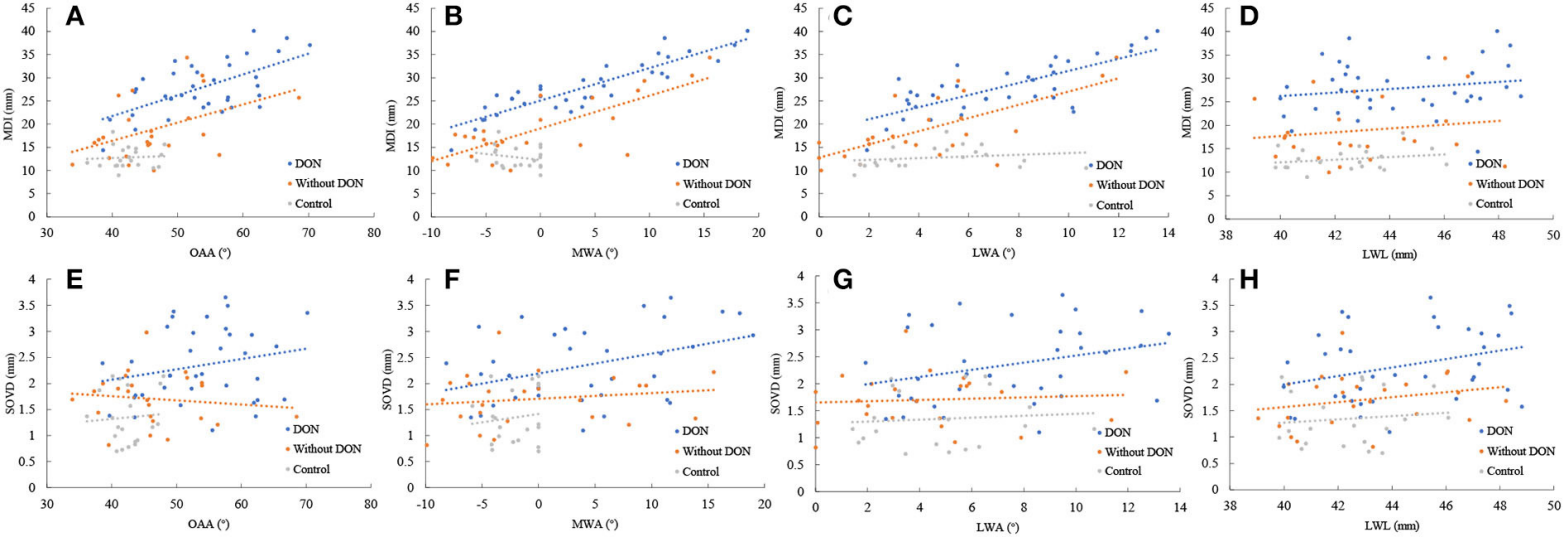
Relationship between the MDI and SOVD with the OAA **(A,E)**, the MWA **(B,F)**, the LWA **(C,G)**, and the LWL **(D,H)** in the orbits with and without DON groups and control group.

Spearman's correlation coefficients were conducted to correlate the bony orbital parameters to the corresponding soft tissue structural parameters (Figure 3). The results showed that the MDI was positively correlated with the MWA in the orbits with the DON group (*r* = 0.877, *p* = 0.000) and without the DON group (*r* = 0.590, *p* = 0.002). The SOVD was positively correlated with the MWA in the orbits with the DON group (*r* = 0.334, *p* = 0.046).

We conducted a multivariate regression analysis with disease status (DON) as the outcome and CT features as independent factors, to identify the predictive factors of DON. The results showed that the MWA (OR 0.629, 95% CI 0.457–0.866, *p* = 0.005), the MDI (OR 1.972, 95% CI 1.326–2.933, *p* = 0.001), and the SOVD (OR 5.113, 95% CI 1.139–22.951, *p* = 0.033) were the independent factors predictive of DON. The regressive equation was obtained as follows: *Y* = −18.171–0.464 MWA + 0.679 MDI + 1.632 SOVD.

**Figure 4:**
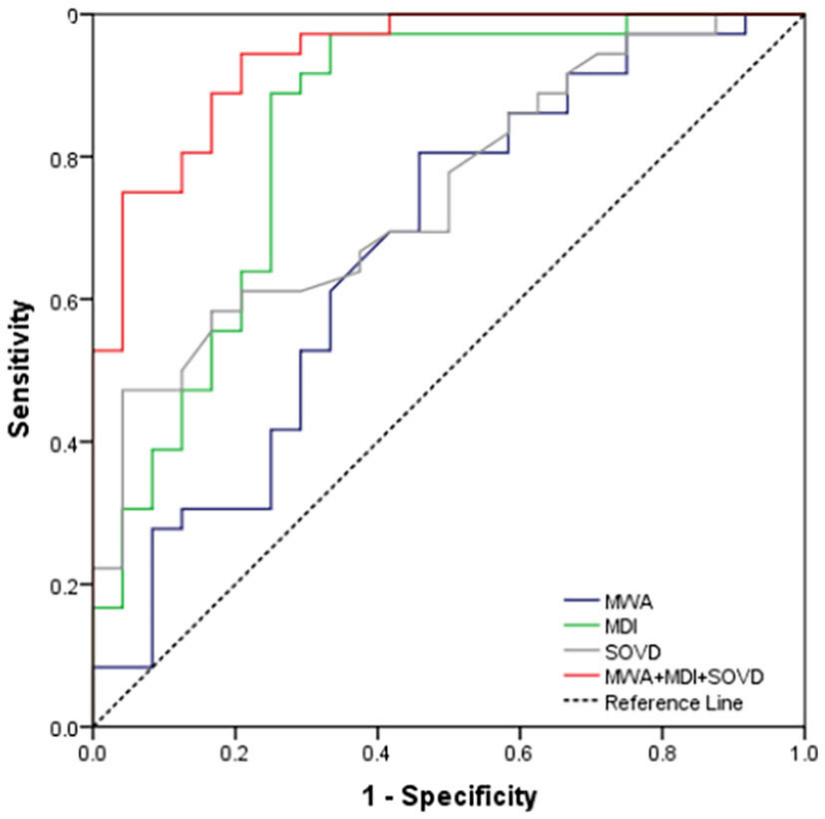
ROC curve for MWA alone, MDI alone, SOVD alone, and combining the above three as a predictor for the occurrence of DON.

Receiver operating characteristic curve analysis demonstrated that the sensitivity and the specificity for the presence of DON by using MWA alone, MDI alone, and SOVD alone were 61% and 67%, 89% and 75%, and 61% and 79%, respectively. Combining the abovementioned three factors simultaneously improved the sensitivity to 89% and the specificity to 83% (Figure 4).

## Discussion

Sight-threatening GO mainly includes DON and exposed corneal ulcers, in which DON is more common clinically. As for the typical clinical manifestations of DON, impaired vision and color vision, as well as abnormalities in the visual field and visual evoked potential tests, could help to indicate DON in patients with GO. However, the radiological findings, especially the orbital cavity and intraorbital soft tissue measurement parameters from the CT scans, could bring benefits for the diagnosis and prediction of DON.

In this study, we determined that the bony orbital angles were larger in the orbits with DON than in the orbits without DON, and the EOM enlargement, the SOV dilatation, and the apical crowding were more severe in the orbits with DON compared with the orbits without DON. The MWA, MDI, and SOVD were the independent factors predictive of DON. The sensitivity and specificity for the presence of DON by using MDI alone and SOVD alone were 89% and 75%, and 61% and 79%, respectively. Including the MWA simultaneously improved the sensitivity to 89% and the specificity to 83%. Our evidence suggests that the MWA combined with MDI and SOVD could better predict the risk of DON in patients with GO. Predictive indicators for the potential presence of DON in patients with GO would offer physicians the ability to early diagnose and design treatment plans and counsel the patient regarding the outcome of the examination.

We sought to characterize the bony orbital and soft tissue structures in existing quantitative measurements on a standard clinical reporting workstation in this study. As the height of the orbital apex was usually larger than its width, the main bony constraint of the increased intraorbital pressure was located in the axial plane rather than in the sagittal plane, so we investigated the angular capacity of the orbital apex in the axial plane. The ORA was used as a reliable bone reference point by Chan et al. (21), and the same approach was used in this study. The mean ORA showed no significant statistical differences between the GO and control groups (*p* = 0.461), indicating that it was effective for us to choose the ORA as the bony reference point.

Sinus cavity gasification changes significantly, and well-gasified sinoids could easily invade the orbit, thus greatly reducing the orbital volume. The orbital medial wall was the thinnest orbital wall, which easily shifted to the medial midline in the presence of increased intraorbital pressure, creating space for increased soft tissue volume in patients with GO and forming spontaneous orbital decompression. Compared with the thicker lateral wall, the medial wall was less resistant to pressure remodeling. Tan et al. (10) demonstrated the existence of orbital bone remodeling by studying the angle of the inferomedial orbital strut and the angle of the medial wall in patients with GO. Accordingly, our study expounded that the changes in the MWA were greater than the LWA in patients with GO. In contrast, the changes in MWA in the normal controls were smaller and always smaller than the ORA. The mean bony orbital angles in the orbits with DON were wider than in the orbits without DON. Likewise, mean EOM diameters were larger in the orbits with DON than in the orbits without DON.

The EOM was the primary occupant of the orbital apex in patients with GO, so the OAA was measured in the plane of the medial rectus and lateral rectus muscles, which we believed to be the plane applying maximum bone remodeling pressure. The orbital apex was defined as the anterolateral border of the groove in the sphenoid body formed by the terminal intra-cavernous portion of the internal carotid artery. The results of this study showed that the OAA in the orbits with the DON group was significantly greater than that in the orbits without the DON group (*p* = 0.001).

Weis et al. (11) divided the medial wall contour into concave, flat, and convex, showing that the OAA and intraorbital wall contour were not related to the occurrence of DON, and only the size of the medial rectus muscle was the most important quantitative predictor of DON. Bokman et al. (23) measured medial wall curvature in 112 orbital CTs of patients with GO and found that significant medial wall curvature existed in 11.6% of GO orbital CTs, and patients with medial wall curvature were more susceptible to DON. However, this study found that the medial rectus muscle diameter and OAA were not related to the occurrence of DON, while the MDI and orbital MWA were predictors of DON. From the multivariate logistical regression analysis in our study, the risk of DON increased with increasing MDI and SOVD. However, when the MWA increases, the risk of DON decreases. Understanding this relationship helps ophthalmologists plan orbital decompression surgery plan.

Meanwhile, the Spearman correlation analysis also showed that bony orbital angles were positively correlated with MDI both in the orbits with and without DON groups, which was most pronounced in the MWA. The results were consistent with the findings of Chan et al. (21), who considered that a greater MDI produced a greater bone remodeling pressure, thus exhibiting a wider bone orbital angle. Notably, for the same MDI, the bony orbital angle was narrower in the orbits with DON than in the orbits without DON. In contrast, for the same bony orbital angle, the MDI was larger in the orbits with DON than in the orbits without DON. We speculated that patients with DON might have a congenital narrow orbit or thicker orbital walls, thus yielding less remodeling pressure, or the onset of DON was more acute and did not have a sufficient bone response, or muscle thickening was too severe to exceed the compensatory limit of bony remodeling.

The mean length of the lateral wall was larger in the orbits with DON (*p* = 0.013), indicating a longer and deeper bony orbital structure in the orbits with DON. On the contrary, the orbits with this structure might be more prone to DON. Moreover, the distance of MRIZL in the orbits with DON was farther than that in the orbits without DON (*p* = 0.019), indicating that the EOM enlargement is the nearer orbital apex in the orbits with DON, further supporting the mechanical compression theory of one of the pathogenesis of DON.

The SOV was valvular-free and was directly connected to the cavernous sinus. The main causes of SOV expansion mainly included internal carotid-cavernous sinus fistula, orbital apex inflammation, orbital inflammatory pseudotumor, and thyroid eye disease. Orbital apical crowding and increased intraorbital pressure in patients with GO might lead to SOV expansion (24). Therefore, in our study, the SOV diameter in the orbits with DON was significantly larger than in the orbits without DON (*p* = 0.000).

Multiple studies have found that the orbital apical crowding score could serve as a good indicator for the diagnosis of DON (16, 19, 20, 25). Our results also showed that the orbital apical crowding score was significantly higher in the orbits with the DON group than in the orbits without the DON group. However, it was not an independent predictor in our multivariate logistical regression analysis. Studies have reported that orbital fat was highly edematous and hyperplastic in patients with GO, increasing the volume of orbital soft tissue and leading to proptosis or compression of the orbital apical optic nerve but no obvious EOM or signs of orbital apical crowding (16, 18, 26). It was not sufficient to predict the risk of DON solely based on EOM or orbital apical crowding score. For increasing the same volume, tight muscle tissue would produce greater remodeling pressure than loose adipose tissue. Chan et al. (21) argued that bony remodeling could compensate for increased intraorbital pressure and was protective. Our study confirmed that the risk of DON decreased when the MWA increased (OR 0.629, *p* = 0.005). We speculated that patients with GO who only exhibited increased fat volume were more likely to develop into DON if their bony orbit was naturally narrow. Further volumetric studies linking intraorbital fat and EOM to the bony orbit may confirm the validity of this argument.

Birchall et al. (19) first proposed that intracranial fat prolapse could serve as one of the indicators of DON, while other studies suggested that this imaging feature was not a reliable risk factor for DON (21, 24). Although intraorbital fat prolapse was more common in orbits with the DON group (50% vs. 41.7%), there was no statistically significant difference in our study, suggesting that patients with GO may already have intracranial fat prolapse before developing DON. In some of the DON cases, orbital imaging showed minimal or no apical crowding but instead increased orbital fat volume and marked proptosis with stretching of the optic nerve and tenting of the posterior pole of the tethered eye. In our study, the optic nerve stretch was the heaviest in the orbits with the DON group (*p* = 0.000), but it was not an independent predictor in our multivariate logistical regression analysis. It was reasonable to consider that the increased soft tissue volume could lead to increased orbital pressure. As the intraorbital content volume and pressure increased, the optic nerve developed a gradual but sustained strain, leading to stretching of the optic nerve, which subsequentially resulted in optic neuropathy. Radial stretching of the optic nerve can lead to indirect circumferential compression of the optic nerve by circumferential tension on the optic nerve sheath (27, 28). All nerves have a critical tensile strength, beyond which axons or blood supply may shear (29). A study attempted to challenge stretch as a mechanism, but the subjects in the study were primarily compressive cases rather than the specific subcategory without EOM enlargement (30).

However, this study also had some limitations. First, it was a retrospective, non-randomized study, which could result in incomplete and inaccurate data. However, the measurement of the orbital CT was objective, and the image study was verified by the intraclass correlation coefficient, which was computed to assess interrater and intrarater variability. Second, the sample size was relatively small. Owing to the relative infrequency of DON, the number of cases was relatively small. Third, the data we used were from a single center and a population of Asian ethnicity. Some of the results might not apply to other ethnic groups. It is more recommended to design a large, multicenter, prospective, randomized, and comparative clinical trial.

## Conclusion

Our study highlighted the importance of both soft tissue structure and bony orbit as predictors of DON. We determined the MWA, the MDI, and the SOVD were the independent factors predictive of DON. Using the abovementioned three indicators could simultaneously improve the sensitivity to 89% and the specificity to 83% for predicting the presence of DON. This approach can help in the risk stratification of the DON. Patients with a larger MDI and SOVD, and relatively narrow medial walls, should be given closer ophthalmic monitoring for early interventions.

## Data availability statement

The raw data supporting the conclusions of this article will be made available by the authors, without undue reservation.

## Ethics statement

The studies involving human participants were reviewed and approved by the Ethics Committee of Union Hospital Affiliated to Tongji Medical College, Huazhong University of Science and Technology, Wuhan, China. The patients/participants provided their written informed consent to participate in this study.

## Author contributions

SC and YM participated in the study design, data analysis, interpretation, performed the statistical analysis, and drafted the manuscript. SC and XW collected the imaging data. YM, MH, YZ, FJ, and XW contributed to the editing and review of the manuscript. ZX and XW contributed to the study design. All authors read and approved the submitted version.

## Funding

This study was supported by the National Natural Science Foundation of China (No. 81900912). The funding organization had no role in the design or conduct of this study.

## Conflict of interest

The authors declare that the research was conducted in the absence of any commercial or financial relationships that could be construed as a potential conflict of interest.

## Publisher's note

All claims expressed in this article are solely those of the authors and do not necessarily represent those of their affiliated organizations, or those of the publisher, the editors and the reviewers. Any product that may be evaluated in this article, or claim that may be made by its manufacturer, is not guaranteed or endorsed by the publisher.
